# The prevalence of myofascial trigger points in neck and shoulder-related disorders: a systematic review of the literature

**DOI:** 10.1186/s12891-018-2157-9

**Published:** 2018-07-25

**Authors:** Daniel Cury Ribeiro, Angus Belgrave, Ana Naden, Helen Fang, Patrick Matthews, Shayla Parshottam

**Affiliations:** Centre for Health, Activity and Rehabilitation Research (CHARR), School of Physiotherapy – University of Otago, PO Box 56, Dunedin, 9054 New Zealand

**Keywords:** Shoulder pain, Neck pain, Trigger point, Myofascial pain, Trigger point, Rotator cuff muscle

## Abstract

**Background:**

Neck and shoulder disorders may be linked to the presence of myofascial trigger points (MTrPs). These disorders can significantly impact a person’s activities of daily living and ability to work. MTrPs can be involved with pain sensitization, contributing to acute or chronic neck and shoulder musculoskeletal disorders. The aim of this review was to synthesise evidence on the prevalence of active and latent MTrPs in subjects with neck and shoulder disorders.

**Methods:**

We conducted an electronic search in five databases. Five independent reviewers selected observational studies assessing the prevalence of MTrPs (active or latent) in participants with neck or shoulder disorders. Two reviewers assessed risk of bias using a modified Downs and Black checklist. Subject characteristics and prevalence of active and latent MTrPs in relevant muscles was extracted from included studies.

**Results:**

Seven articles studying different conditions met the inclusion criteria. The prevalence of MTrPs was compared and analysed. All studies had low methodologic quality due to small sample sizes, lack of control groups and blinding. Findings revealed that active and latent MTrPs were prevalent throughout all disorders, however, latent MTrPs did not consistently have a higher prevalence compared to healthy controls.

**Conclusions:**

We found limited evidence supporting the high prevalence of active and latent MTrPs in patients with neck or shoulder disorders. Point prevalence estimates of MTrPs were based on a small number of studies with very low sample sizes and with design limitations that increased risk of bias within included studies. Future studies, with low risk of bias and large sample sizes may impact on current evidence.

**Electronic supplementary material:**

The online version of this article (10.1186/s12891-018-2157-9) contains supplementary material, which is available to authorized users.

## Background

Neck and shoulder pain are common complaints that may significantly impact a person’s activities of daily living and their ability to work [1]. In New Zealand, shoulder pain is the third most common musculoskeletal condition. Neck pain is the 4th highest condition in terms of years lived with disability [2]. Within New Zealand, ACC reports the 12-month prevalence estimates for neck pain in the adult population lie between 30 and 50%, and accounts for 15% of the global burden of disease [2].

Myofascial trigger points (MTrPs) are considered to be hypersensitive, tender areas over a taut band of muscle [3]. They are palpable, produce localised and referred pain to other structures with mechanical stimulation [4, 5]. MTrPs can be further differentiated as active or latent [3]. Active and latent MTrPs elicit local and referred pain, however active MTrPs also reproduce patient symptoms, whereas latent MTrPs do not [3, 4, 6]. Latent MTrPs may later become active [3, 7]. It is considered that both active and latent MTrPs can cause muscle imbalances, weakness and impaired motor recruitment, disrupting muscle function, and exposing joint to suboptimal loading [8].

The theory of trigger points causing myofascial pain syndrome is controversial with limited external validity to support it [9]. Despite that, physiotherapy interventions commonly target MTrPs [10, 11]. Active and latent MTrPs may contribute to neck and shoulder pain symptoms [12]. In a study with small sample size, active MTrPs were found to present higher concentration of inflammatory mediators, neuropeptides, cytokines, and catecholamines if compared to latent MTrPs or other body regions with no MTrPs [13]. Latent MTrPs were found to impact on motor recruitment patterns [14], accelerate fatigue [15] in agonist muscles, and seem to be linked to increased muscle activity of antagonist muscles [16].

Patients with chronic, non-traumatic neck and shoulder pain were found to have higher prevalence of MTrPs when compared to healthy controls, with different distributions between muscles of two opposing anatomical structures [12]. For example, active MTrPs were prevalent in the infraspinatus and upper trapezius muscles, whilst latent MTrPs were prevalent in the teres major and anterior deltoid muscles [12]. Studies investigating shoulder impingement have reported active MTrPs in infraspinatus, subscapularis, supraspinatus, and pectoralis major muscles [17]. Together, these findings suggest that MTrPs are likely to be present in different shoulder and neck disorders, and may vary in muscle distribution and type (i.e. active or latent).

Knowledge of MTrP common locations at the neck and shoulder can help clinicians to optimally prescribe interventions to manage neck and shoulder disorders. To our knowledge no previous systematic review summarized findings from and assessed the methodological quality of studies assessing the prevalence of MTrPs in the upper quadrant (i.e. neck and shoulder disorders). A previous review focused on spinal disorders only, without assessing patients with shoulder disorders [18]. Given the link between these structures (i.e. neck, scapula and shoulder), we deemed appropriate to conduct a systematic review assessing the upper quadrant. Therefore, the objective of this study was to synthesize the current evidence on the prevalence of active and latent MTrPs in subjects with acute or chronic neck and shoulder disorders. The specific aims were to: (1) identify the prevalence of MTrPs in neck, scapular and shoulder muscles; and (2) compare the prevalence of MTrPs in subjects with diagnosed acute or chronic neck or shoulder-related disorders to healthy controls.

## Methods

*The protocol of this review is described in* Additional file 1*.*

### Study retrieval and screening

A comprehensive literature search of databases including CINAHL, Embase, Pubmed, Scopus and Web of Science was completed on August 12, 2017. The search strategy used is presented in Table 1. Screening of reference lists from included studies was also performed. Articles were then exported into Endnote and duplicates were removed. The retrieved articles were screened for eligibility by title, followed by full-article screening. There was no attempt to access unpublished studies or supplementary ‘grey’ literature.

**Table 1:** Search strategy and key terms used

Database	Keywords	Number of Studies
CINAHL	(1) shoulder/ or glenohumeral/ or scapular/ or scapula/ or neck/ or cervical; (2) trigger point/ or trigger points; (3) prevalence; (4) disease/ or musculoskeletal diseases; (5) 1 and 2 and 3 and 4	3
Embase	(1) shoulder/ or glenohumeral/ or scapular/ or scapula/ or neck/ or cervical; (2) trigger point/ or trigger points; (3) prevalence; (4) disease/ or musculoskeletal diseases; (5) 1 and 2 and 3 and 4	2
Pubmed	(1) shoulder/ or glenohumeral/ or scapular/ or scapula/ or neck/ or cervical; (2) trigger point/ or trigger points (3) prevalence (4) “disease” [MeSH Terms]/ OR disease [All Fields]/ or disorder [All Fields]/ or condition [All Fields]/ or “musculoskeletal diseases” [MeSH Terms] (5) 1 and 2 and 3 and 4	40
Scopus	(1) shoulder/ or glenohumeral/ or scapular/ or scapula/ or neck/ or cervical; (2) trigger point/ or trigger points; (3) prevalence; (4) disease/ or musculoskeletal diseases; (5) 1 and 2 and 3 and 4	30
Web of Science	(1) shoulder/ or glenohumeral/ or *scapula*/ or neck/ or Cervical; (2) trigger* point*; (3) prevalence; (4) disease/ or musculoskeletal diseases; (5) 1 and 2 and 3 and 4	38
Total number of articles identified Excluding Duplicates		113 84

Initially, two independent reviewers screened articles by titles, and a third reviewer was available if consensus was not achieved. Full texts of potential eligible studies were retrieved and assessed independently against the inclusion criteria by two reviewers (A.B., and P.M.). Discrepancies between reviewers regarding full text eligibility were resolved in a consensus meeting and a third reviewer (DCR) was consulted.

### Eligibility criteria

The following study designs were included in this review: (1) full-text articles published in a peer-reviewed scientific journal; (2) observational, cross-sectional, or prospective studies assessing the prevalence of active and/or latent MTrPs in at least one group of adult subjects (> 18 years old) with a shoulder, scapular, or neck disorder (as diagnosed by the original study); and (3) inclusion of manual assessment of MTrPs in at least one specific neck, scapular or shoulder muscle. Articles in any languages and medical diagnoses indicating the presence of shoulder, scapular, or neck were accepted and included in this review. All study designs other than the aforementioned were excluded, unless randomised control trials included the prevalence of MTrPs as a baseline measurement.

### Risk of bias within included studies

A modified Downs and Black checklist [19] was used to assess the risk of bias within included studies. This checklist is a 27 item checklist; however, 11 items were excluded as these were not applicable for this systematic review. Each study was assessed independently by 2 reviewers. Disagreements were resolved through consensus, if consensus was not reached, then a third author (D.R) was consulted. Studies scoring 50% or more were considered to have low risk of bias; whilst studies presenting with a Downs and Black score lower than 50% were considered to have a high risk of bias. For the purpose of this review, we arbitrarily selected a 50% cut-off. This threshold been used by a previous systematic review assessing observational studies [18].

### Data extraction

Characteristics from each study and additional patient and control group information were extracted and recorded. The proportion of participants with active/latent MTrPs in all assessed muscles were documented from each study. When available, we extracted information regarding the duration of the condition (i.e. acute or chronic). The data was independently extracted by two reviewers, and double-checked for accuracy.

### Data analysis

As each included study analysed a different disorder, it was not possible to conduct a meta-analysis. Therefore, a narrative discussion of findings is presented.

## Results

**Fig. 1:**
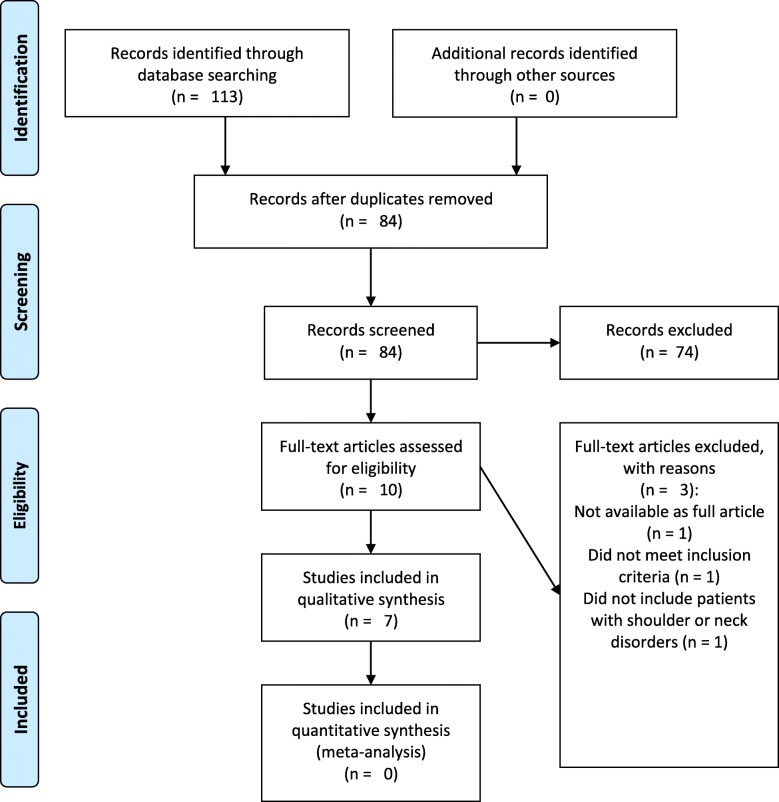
Study Selection

The flow of studies in the review is presented in Fig. 1. Seven articles were included in this systematic review, with a sample size of 433 participants. Four studies had a cross-sectional design, while three studies used a case-control design.

### Risk of bias within included studies

The risk of bias within included studies is presented in Table 2. Overall, studies were considered as having low risk of bias. There is some risk of bias for external validity. For example, subjects were considered as not representative of the entire population in three studies, and it was not possible to determine this in two studies (Item 8, Table 2). Risk of bias for internal validity was mainly due to participants not being recruited from the same population (Item 14 – Table 2) or due to lack of clarity about the recruitment period (not clear in four studies – Item 15, Table 2). Finally, three studies did not estimate sample size a priori.

**Table 2:** Risk of bias within included studies

Study	Reporting Bias							External Validity			Internal validity					Power	Risk of Bias
	Item 1	Item 2	Item 3	Item 4	Item 5	Item 6	Item 7	Item 8	Item 9	Item 10	Item 11	Item 12	Item 13	Item 14	Item 15	Item 16	
Alonso-Blanco et al., 2011	✓	✓	✓	✓	✓	✓	✓	✓	✗	✗	✓	✓	✓	✓	✓	✗	Low
Bron et al., 2011	✓	✓	✓	✓	✓	✓	✗	✓	✓	✗	✓	✓	✓	✓	✓	✓	Low
Fernández-De-Las-Peñas, 2012	✓	✓	✓	✓	✓	✓	✓	✗	?	✓	✓	✓	✓	✗	?	✓	Low
Fernandez-Perez, 2012	✓	✓	✓	✓	✓	✓	✓	✗	✓	✓	✓	✓	✓	✗	?	✓	Low
Hilalgo-Lozano et al., 2010	✓	✓	✓	✓	✓	✓	✓	?	?	✓	✓	✓	✓	✗	?	✗	Low
Sari et al., 2012	✓	✓	✓	✗	✓	✓	✓	?	?	✗	✓	✓	✓	?	?	✗	Low
Tali et al., 2014	✓	✓	✓	✓	✓	✗	✓	✗	✓	✓	✓	✓	✓	✓	✓	?	Low

Abbreviations: ✓, yes ✗, no?, unable to determine

### Characteristics of included studies

The included studies analysed the following disorders: chronic tension-type headache, chronic non-traumatic unilateral shoulder pain, non-specific upper quadrant pain, acute whiplash disorder, unilateral shoulder impingement syndrome and episodic migraine. For each disorder, MTrP prevalence was assessed in different muscles (e.g. upper trapezius, supraspinatus, sternocleidomastoid) (Table 3). The characteristics of the subjects included in the studies is presented in Table 4, and the point prevalence of active MTrPs in subjects with shoulder or neck disorders is presented in Table 5.

**Table 3:** Characteristics of included studies

Study	Study design	Disorder(s)	Healthy Controls Group	Diagnostic Criteria Active MTrPs	Diagnostic Criteria Latent MTrPs	Assessed Muscles	Country, Setting
Alonso-Blanco et al., 2011	Cross-sectional	CTTH	No	1) Presence of a palpable taut band in a skeletal muscle. 2) Presence of a hyperirritable sensitive spot within the taut band. 3) Local twitch response elicited by the snapping palpation of the taut band. 4) Presence of referred pain in response to MTrP compression.	Not assessed	Upper Trapezius Sternocleidomastoid Temporalis Suboccipital	Spain, hospital
Bron et al., 2011	Cross-sectional	SP	No	1) A nodule in a taut band of skeletal muscle. 2) Painful on compression 3) May produce referred pain or sensations 4) Pain recognised by patient as “familiar”	1) A nodule in a taut band of skeletal muscle. 2) Painful on compression 3) May produce referred pain or sensations 4) Pain not recognizable to patient	Upper/middle/lower trapezius Infraspinatus Supraspinatus Subscapularis Teres minor and major Anterior/middle/posterior deltoids Pectoralis major and minor Biceps brachii Triceps brachii Scalene Subclavius	Spain, primary care practice.
Fernandez-Perez et al., 2012	Cross-sectional cohort	Acute WAD	Yes	1) Palpable taut band within a skeletal muscle 2) Presence of a hypersensitive spot in the taut band 3) Local twitch re-sponse elicited by the snapping palpation of the taut band 4) Production of referred pain in response to MTrP manual compression. 5) If referred pain of symptoms reported by the patient is recognized as familiar	1) Palpable taut band within a skeletal muscle 2) Presence of a hypersensitive spot in the taut band 3) Local twitch re-sponse elicited by the snapping palpation of the taut band 4) Production of referred pain in response to MTrP manual compression. 5) Symptoms produced are not familiar to the patient	Temporalis Masseter Upper trapezius Levator scapulae Sternocleidomastoid Scalene	Spain, primary care
Fernández-De-Las-Peñas, 2012	Cross-sectional	Non-specific pain	No	1) Presence of a palpable taut band within a skeletal muscle. 2) Presence of a hyperirritable spot in the taut band. 3) Local twitch response elicited by the snapping palpation of the taut band (when possible). 4) Presence of referred pain in response to compression. MTrPs were considered active when the local and referred pains evoked by compression reproduced clinical pain symptoms and also the participant recognized the pain as familiar.	MTrPs were considered latent when the local and the referred pain elicited by digital compression did not reproduce symptoms familiar to the participant.	Temporalis Masseter Upper trapezius Sternocleidomastoid Splenius capitis Oblique capitis inferior Levator scapulae Scalene Pectoralis major Deltoid Infraspinatus Extensor carpi radialis brevis Extensor carpi radialis longus Eetensor digitorum communis Supinator	Spain, Department of PT, OT, rehab and physical medicine.
Hidalgo-Lozano et al., 2010	Case-control	Unilateral shoulder impingement	Yes	1) Presence of a palpable taut band in a skeletal muscle 2) Presence of a hyperirritable tender spot within the taut band 3) Local twitch response elicited by the snapping palpation of the taut band 4) Presence of referred pain in response to MTrP compression. 5) Local and the referred pain evoked by digital compression reproduced the pain symptoms (both in location and pain sensation) and the subject recognized the pain as familiar pain	1) Presence of a palpable taut band in a skeletal muscle 2) Presence of a hyperirritable tender spot within the taut band 3) Local twitch response elicited by the snapping palpation of the taut band 4) Presence of referred pain in response to MTrP compression. 5) Local and referred pain elicited by digital compression did not reproduce symptoms familiar to the subjects	Levator scapulae Supraspinatus Infraspinatus Subscapularis Pectoralis major Biceps brachii	Spain, setting unclear
Sari et al., 2012	Case-control	Cervical Radiculopathy	Yes	1) Presence of a palpable taut band in a skeletal muscle 2) Presence of hypersensible tender spot in the taut band 3) Local twitch response elicited by the snapping palpation of the taut band 4) Reproduction of the typical referred pain pattern of the MTrP in response to compression; and 5) Spontaneous presence of the typical referred pain pattern and/or patient recognition of the referred pain as familiar. If all of the aforementioned criteria were present the MTrP was considered active	1) Presence of a palpable taut band in a skeletal muscle 2) Presence of hypersensible tender spot in the taut band 3) Local twitch response elicited by the snapping palpation of the taut band 4) Reproduction of the typical referred pain pattern of the MTrP in response to compression	Trapezius, multifidus, splenius capitis, levator scapulae, rhomboid major, and rhomboid minor	Turkey, Outpatient clinic
Tali et al., 2014	Case-control	Episodic migraines	Yes	1) Palpable taut band within a skeletal muscle 2) Presence of a hypersensitive spot in the taut band 3) Local twitch response elicited by the snapping palpation of the taut band 4) Production of referred pain in response to MTrP manual compression. 5) If the MTrP were palpated and produced a headache, familiar or not, it was referred to as an “active MTrP”.	1) Palpable taut band within a skeletal muscle 2) Presence of a hypersensitive spot in the taut band 3) Local twitch response elicited by the snapping palpation of the taut band 4) Production of referred pain in response to MTrP manual compression. 5) If the MTrP were palpated and produced local or radiated pain it was referred to as a “latent MTrP”.	Sternocleidomastoid Upper trapezius	Israel, Physiotherapy Department

Abbreviations: *CTTH* Chronic tension type headache, *SP* Shoulder pain, *WAD* Whiplash associated disorder, *MTrP* Myofascial trigger point

**Table 4:** Characteristics of the subjects included in the studies

Study	Stage and type of disorder	Patients Sample, n	Sex, % Female	Age (y), Mean ± SD or %	Other Characteristics, Mean ± SD or %	Healthy Controls Sample, n	Sex, % Female	Age (y), Mean ± SD or %	Other Characteristics, Mean ± SD or %
Alonso-Blanco et al., 2011	Chronic TTH	20 Children (6–12 years) 20 Adults (18–47 years)	50% female	Children = 8 ± 2 years Adults = 41 ± 11 years	PH(years)- Children = 1.6 ± 0.8 Adults = 8.6 ± 6.5 HI (NPRS)- Children = 5.0 ± 1.2 Adults = 5.9 ± 1.1 HD (hours/days)- Children = 4.8 ± 2.6 Adults = 7.3 ± 2 HF(days/week)- Children = 4.0 ± 0.9 Adults = 4.3 ± 0.9	0	Not assessed	Not assessed	Not assessed
Bron et al., 2011	Chronic SP	72 patients	69% Female	43.9 ± 12.3 years	Duration- 6–9 months = 23% 9–12 months = 19% 1–2 years = 18% 2–5 years = 19% > 5 years = 19% Recurrence rate- 1st = 36% 2nd = 26% 3rd > = 37%	0	Not assessed	Not assessed	Not assessed
Fernandez-Perez et al., 2012	Acute WAD	20 participants aged over 20 years	50% women	28.7 ± 12.4 (22.9,34.4)	Height, cm: 170.0 ± 10.6 (165.0,175.0) Weight kg: 67.7 ± 16.3 (60.1,75.4) Time from accident, d: 26.6 ± 3.8 (24.8,28.4) Current pain (NPRS): 6.2 ± 2.6 (5.0, 7.5) Worst pain (NPRS): 8.0 ± 2.0 (7.0, 8.9) Lowest pain (NPRS): 3.3 ± 2.9 (1.9, 4.7)	20	50% female	29.1 ± 12.2 (23.3, 34.8)	Height cm: 160.7 ± 39.1 (142.3, 179.0) Weight kg: 64.1 ± 23.3 (53.2, 75.1)
Fernández-De-Las-Peñas, 2012	NSP	16 Blue collar workers 19 White collar worker	Blue = 62% Female White = 75% female	Blue = 44 ± 13 years White = 44 ± 14 years	PH (months)- Blue = 13.2 ± 5.3 White = 9.1± 5.5 MP (NPRS)- Blue = 5.0 ± 2.5 White = 3.8 ± 2.6	0	Not Assessed	Not Assessed	Not Assessed
Hidalgo-Lozano et al., 2010	ULSIStage of condition unclear	12 patients	42% female	25 ± 9 years		10	50% female	26 ± 8 years	
Sari 2012	Acute and chronic Cervical radiculopathy	244	52% female	44.6 ± 10.3 years	BMI (kg/m^2^)- 26.28 ± 5.25 Social status: Married 70%, single 23%, widow 7% Education: University 40%, high school 38%, primary education 18%, uneducated 4% Occupation: Housewife 32%, worker 25%, retired 13%, student 9%, officer 8%, nurse 5%, other 8% Major trauma history 15.6%	122	N/A	43.8 ± 9.8 years	N/A
Tali et al., 2014	EM Stage unclear	20 Physical therapy students	90% female	24.95 ± 1.79 years	BMI (kg/m^2^): 21.68 ± 2.62 HF (days in the past 3 months): 6.60 ± 5.88 AS (NPRS): 6.45 ± 1.50	20 Physical Therapy students	85% female	25.65 ± 1.42 years.	BMI (kg/m2)- 21.69 ± 2.08

Abbreviations: *TTH* tension type headache, *SP* Shoulder pain, *WAD* Whiplash associated disorder, *NSP* Non-specific pain, *EM* Episodic migraines, *ULSI* Unilateral shoulder impingement, *SD* Standard deviation, *PH* Pain history, *HI* Headache intensity, *HD* Headache duration, *HF* Headache frequency, *AS* Average severity, *NPRS* Numeric pain rating scale, *MP* Mean pain, *BMI* Body mass index

**Table 5:** Point prevalence (Expressed as percentage) of active MTrPs in subjects with shoulder or neck disorders

	Alonso-Blanco et al., 2011		Bron et al., 2011	Fernandez-Perez et al., 2012	Fernández-De-Las-Peñas, 2012		Hidalgo-Lozano et al., 2010	Sari, 2012	Tali et al., 2014	Hidalgo-Lozano et al., 2010
Sample	Adults (*N* = 20)	Children (*N* = 20)			White collar (*N* = 19)	Blue collar (*N* = 16)				
Information regarding right or left side			Do not specify left and right				Do not specify left and right	Do not specify left and right		Do not specify left and right
Muscles										
Left Temporalis	55	70		20	5.3	6.3				
Right Temporalis	65	75		10	5.3	6.3				
Right SCM	30	25		5	21.1	6.3			5	
Left SCM	40	10		30	21.1	18.8			10	
Left Upper Trapezius	35	20		30	63.2	56.3		13.5	25	
Right Upper Trapezius	80	15		35	63.2	68.8			45	
Suboccipital muscles	100 (bilateral)	80 (bilateral)			OCI- left = 31.6. right = 31.6 SC- left = 15.8. right = 21.1	OCI- left = 12.5. right = 25 SC- left = 31.3. right = 37.5				
Middle Trapezius			43.1							
Lower Trapezius			37.5							
Left Infraspinatus			77.8		31.6	37.5	41.7			41.7
Right Infraspinatus					21.1	43.8				
Supraspinatus			34.7				66.7			66.7
Subscapularis			40.3				41.7			41.7
Teres minor			47.2							
Teres major			36.1							
Left Deltoid			Posterior- 44.4 Middle- 50 Anterior- 47.2		5.3	18.8				
Right Deltoid					10.5	12.5				
Left Pectoralis major			26.4		5.3	18.8	16.7			16.7
Right Pectoralis major					18.8	18.8				
Pectoralis minor			30.6							
Biceps Brachii			20.8				16.7			16.7
Triceps Brachii			19.4							
Left Scalene			16.7	20	21.1	12.5				
Right Scalene				30	15.8	2.3				
Subclavius			25							
Left Masseter				10	15.8	0				
Right Masseter				0	5.3	0				
Left Levator scapulae				55	31.6	25	41.7	16.3		41.7
Right Levator scapulae				65	36.8	12.5				
Splenius capitis								14.7		
Rhomboid minor								14.3		
Rhomboid major								10.2		
Multifidus								8.6		

Abbreviations: CTTH – Chronic tension type headache SP – Shoulder pain, WAD – Whiplash associated disorder, NPRS – Numerical pain rating scale, OCI – Oblique capitis inferior, SC – Splenius capitis, SCM -Sternocleidomastoid

### Chronic tension-type headache

Alonso-Blanco et al. (2011) analysed the prevalence of active MTrPs in dominant and non-dominant temporalis, upper trapezius, sternocleidomastoid (SCM), and bilateral suboccipital muscles [5]. The sample contained 20 adults and 20 children with chronic tension-type headache. Results showed adults had a significantly (*p* = 0.001) higher number of active MTrPs (4, SD = − 0.8) than children (3, SD = − 0.7). Significant differences in the distribution of active MTrPs between adults and children were found in the dominant upper trapezius (*p* < 0.001), and the non-dominant SCM (*p* = 0.032) muscles. This study had some external validity and power bias.

### Chronic non-traumatic unilateral shoulder pain

Bron et al. (2011) reported on the prevalence of MTrPs in 72 subjects with chronic non-traumatic unilateral shoulder pain [20]. This study analysed the prevalence of MTrPs on upper trapezius, middle trapezius, lower trapezius, infraspinatus, supraspinatus, subscapularis, teres minor, teres major, posterior deltoid, middle deltoid, anterior deltoid, pectoralis major, pectoralis minor, biceps, triceps, scalenes and subclavius muscles. Muscles containing active MTrPs were present in all participants, and the median number of MTrPs was 6 (range 2–16) per subject. Latent MTrPs were found in 67 participants with a median of 4 (range 0–11). Active MTrPs were most prevalent in the infraspinatus (*n* = 56) and the upper trapezius muscles (*n* = 42); whereas latent MTrPs were most prevalent in the teres major (*n* = 35), anterior deltoid (*n* = 27) and upper trapezius (n = 27) muscles. Although there was no difference found between left and right sides, this study demonstrated a high prevalence of active and latent MTrPs in muscles of patients with non-traumatic shoulder pain. This study had small reporting and external validity bias.

### Non-specific upper quadrant pain

Fernández-De-Las-Peñas et al. (2012) analysed the prevalence of active MTrPs in the head, neck an d arm between manual (blue collar) and office (white-collar) workers with nonspecific neck or shoulder pain [21]. There was a similar number of MTrPs in the upper quadrant musculature with the most prevalent being upper trapezius, infraspinatus, levator scapulae, and extensor carpi radialis brevis muscles for both groups. No significant difference between groups was found with regards to the distribution of active and latent MTrPs, or the total number (*p* = 0.503) of active (*p* = 0.657) and latent (*p* = 0.605) MTrPs. Manual workers demonstrated a mean of 6 (SD = 3) active MTrPs, and 10 (SD = 6) latent MTrPs compared to the 6 (SD = 4) active and 11 (SD = 5) latent MTrPs shown in office workers [21]. This study had some reporting, external and internal validity risk of bias.

### Acute whiplash disorder

Fernandez-Perez et al. (2012) compared the prevalence of MTrPs in patients with a high level of disability following acute whiplash injuries with healthy controls [22]. The distribution of MTrPs were statistically significant (*p* < 0.05) in the temporalis, upper trapezius, SCM, levator scapulae, scalenes, and suboccipital muscles between patient and healthy control groups. Active MTrPs in levator scapulae (*p* = 0.012) and upper trapezius (*p* < 0.01) muscles were more prevalent in the patient group when compared to healthy controls. When compared to healthy controls participant’s suffering from whiplash associated disorder (WAD) had significantly higher prevalence in the mean total number of MTrPs per person (7.3, SD = 2.8). Patients with WAD had a significantly higher number of active MTrPs per person (3.9, SD = 2.5). No active MTrPs were found in healthy controls (*p* < 0.001). Significant differences in latent MTrPs were also observed between groups (*p* = 0.002). Participants with acute WAD had a mean of 3.4 (SD = 2.7) latent MTrPs per person, whereas healthy subjects had a mean of 1.7 (SD = 2.2) [22]. This study had some reporting, external and internal validity risk of bias.

### Unilateral shoulder impingement syndrome

Hidalgo-Lozano et al. (2010) assessed the prevalence of MTrPs in 12 patients with unilateral shoulder impingement syndrome compared to healthy controls [17]. On average each patient had 4.5 (SD = 1) MTrPs and of those, 2.5 (SD = 1) were active MTrPs and 2 (SD = 1) were latent. However, no distinction was made between left and right shoulders. Point prevalence of active MTrPs was most predominant in supraspinatus (67%), infraspinatus (42%) and subscapularis (42%). The distribution of MTrPs in muscles was also significantly higher in individuals with unilateral shoulder impingement syndrome in comparison to healthy controls. Differences in MTrPs between healthy controls and symptomatic participants were reported for the levator scapulae, supraspinatus, infraspinatus, pectoralis major, and biceps brachii but not subscapularis muscles. Both active and latent MTrPs were present in unilateral shoulder impingement participants with levator scapula (100%), supraspinatus (66%), infraspinatus (83.33%), and subscapularis (66%). This study had some reporting, external and internal validity, and power risk of bias.

### Cervical radiculopathy

One study assessed the presence of active and latent MTrPs in patients with cervical radiculopathy [23]. The muscles assessed included trapezius, multifidus, splenius capitis, levator scapulae, rhomboid major, and rhomboid minor. A total of 244 patients where compared to 122 controls. Findings suggest that active MTrPs are more common on patients with cervical radiculopathy than controls. Participants on the control group did not present active MTrPs on assessed muscles. The study also reported no difference between groups (control and cervical radiculopathy) in the distribution of latent MTrPs (*p* = 0.249). This study had some reporting, external and internal validity, and power risk of bias.

### Episodic migraine

Tali et al. (2014) studied two groups with the first (18 women and 2 men) suffering from episodic migraines and the second (17 women and 3 men) being healthy controls [24]. Results from this study revealed an increased number of active MTrPs in the migraine group when compared to healthy controls. No significant difference (*p* = 0.185) between groups was found for the prevalence of latent MTrPs. That study identified a higher prevalence of MTrPs (active and latent) in the migraine group in the right trapezius in comparison to the control group [24]. There was no significant difference in MTrPs between left and right side migraines. This study had some reporting, external validity, and power risk of bias.

## Discussion

This systematic review aimed to synthesise evidence on the prevalence of active and latent MTrPs in neck or shoulder disorders. Seven studies were included, each study focused on different populations and conditions. All studies scored 9/16 or higher on the modified Downs and Black checklist, suggesting an overall low risk of bias within included studies. We have identified risk of reporting, external and internal validity and power bias in included studies.

The included studies examined the following musculoskeletal disorders: chronic tension-type headache [5], unilateral shoulder pain [20], upper quadrant pain [21], acute whiplash disorder [22], shoulder impingement syndrome [17], cervical radiculopathy [23], and episodic migraine [24]. Hidalgo-Lozano et al. (2010), Fernandes-de-las-Penas et al. (2012), Fernandes-Perez et al. (2012), and Tali et al. (2014) all compared participants with shoulder or neck disorders to healthy controls and found participants with shoulder and neck disorders had a higher prevalence of MTrPs [17, 21, 22, 24]. Active and latent MTrPs are more common in the upper trapezius muscle, with the exception of Fernández-De-Las-Peñas et al. (2012) who found no significant difference between the distribution on active or latent MTrPs [21].

Alonso-Blanco et al. (2011) and Bron et al. (2011) compared the prevalence of MTrPs between adults and children and the prevalence between right and left shoulders in patients with unilateral shoulder pain respectively [5, 20]. Alonso-Blanco et al. (2011) found that adults had higher prevalence of MTrPs than children, whilst in contrast Bron et al. (2011) found no significant differences between symptomatic and asymptomatic shoulders [20].

All studies, with the exception of two [20, 23] had very small samples sizes. Therefore, the generalisability of results is limited. Bron et al. (2011) and Sari et al. (2012) had the two largest sample sizes (72 and 244 patients respectively) from all 7 studies [20, 23]. However, Bron et al. (2011) study had 69% female participants and did not include a healthy control group which decreases the significance of their findings [20]. Bron et al. (2011) and Hidalgo-Lozano et al. (2010) did not differentiate between the prevalence of left and right MTrPs and they did not acknowledge which side was symptomatic [17, 20]. This made interpreting results more difficult and hindered the synthesis of data from multiple studies.

Studies presented limitations regarding sample size and assessor blinding. All studies were considered to have small sample sizes [25]. A small sample size can lead to biased results. Future studies with larger sample sizes should be designed, and estimated a priori, to ensure more reliable and accurate findings. Only four studies ensured practitioners assessing trigger points were blinded. Blinding the assessor helps reducing the influence of the assessors’ perception and believes towards an outcomes measure [26].

Only 4 of the 7 studies included a control group. Findings from control groups inform what outcomes are expected within an asymptomatic population. Additionally, the inclusion of a control group helps to control for other variables (e.g. age, occupation), and also accounts for normal biological variations [27]. Future studies should therefore include a control group, to enhance our understanding on the role of MTrPs and musculoskeletal disorders.

There is lack of consensus on how to define myofascial trigger point pain syndrome [28]. The use of different definitions, or the lack of clarity around MTrPs definition, impact on the external validity of reported findings. Included studies used similar (but not always the same) diagnostic criteria for assessing active and latent MTrPs. Currently, the criterion validity of MTrPs diagnostic criteria is poor, as there is no gold standard for diagnosing MTrP. Therefore, it is unknown what the sensitivity and specificity is when using the clinical criteria proposed by Simons et al. [29].

The reliability of physical examination for diagnosing MTrP has been questioned in the literature [30]. One study reported excellent test-retest reliability for physical examination when assessing MTrPs in patients with rotator cuff disorders [31]. On the other hand, two previous systematic reviews [30, 32] questioned the reliability of physical examination for assessing the presence of MTrP due to low methodological quality of included studies. There is definitely a need for an international consensus for standardizing the assessment of MTrP in clinical practice and research [18].

All studies used very similar definitions to define a MTrP (Table 3). Most used the description from “Myofascial Pain and Dysfunction. The trigger point manual. Upper half of body” by Simons et al. [29] The criteria often comprised*: 1) presence of a palpable taut band within a skeletal muscle; 2) existence of a hyperirritable spot in the taut band; 3) local twitch response elicited by the snapping palpation of the taut band (when possible); 4) presence of referred pain in response to compression* [29]. The criteria for distinguishing between active and latent MTrPs were also defined by Simons et al. [29]. The difference in active and latent MTrPs was found following compression of the MTrP. If patient’s symptoms were reproduced, it is considered to be an active MTrP; whereas no reproduction of symptoms or production of unfamiliar symptoms is considered latent [20].

The results from this review suggest that active and latent MTrPs are highly present in patients with different neck or shoulder disorders. From the 7 included studies, 5 revealed that the upper trapezius was consistently one of the muscles with highest, if not the highest, prevalence of a MTrP. Furthermore, 3 studies examined the prevalence of MTrPs on infraspinatus muscle [17, 20, 21] and, together, these findings suggest that infraspinatus is among one of the most prevalent muscles with active MTrPs across all 3 studies.

All seven studies reported the importance of referred pain mechanism relating to MTrPs, and how it may be an underlying contributing factor to the patient’s condition. Alonso-Blanco et al. (2011) found a significantly higher number of active MTrPs in adults and discussed the similarities observed between the presence of active MTrPs and patterns of their headache symptoms [5]. Hidalgo-Lozano et al. (2011) revealed that the referred pain pattern from the active MTrPs of the levator scapulae, supraspinatus, infraspinatus, subscapularis, pectoralis major, and biceps brachii reproduced patient symptoms [17]. This was also in agreement with Dong et al. (2015) and Koester et al. (2005) who reported shoulder impingement often refers pain down to the mid humerus level, further increasing the validity of MTrPs and their impact on the reported symptoms for this disorder [33, 34]. These studies support the idea that high active MTrPs may contribute to patient’s symptoms.

### Study limitations and previous systematic reviews

We were unable to perform a meta-analysis due to patients with different disorders being included, and different outcome measures used by the included studies. Due to limited number of articles included in this review, we could not explore differences in the prevalence of MTrPs between acute and chronic conditions. We did not register the protocol of this review, and that increases risk of reporting bias of this review. A previous systematic review assessed the prevalence of MTrPs in spinal disorders [18]. Findings from the review support the theory that MTrPs are more prevalent in patients with musculoskeletal disorders.

## Conclusion

Findings from this systematic review suggest that there is limited evidence supporting the high prevalence of active and latent MTrPs in patients with neck or shoulder disorders. Point prevalence estimates of MTrPs were based on a small number of studies with very low sample sizes and with design limitations that increased risk of bias within included studies. Therefore, future studies assessing patients neck or shoulder disorders, with large samples and stronger study designs are required to provide more reliable pooled estimates of point prevalence of MTrPs in these patients.

## Additional file

Additional file 1: Protocol. (DOCX 94 kb)
