# Supplementary material for: The prevalence of myofascial trigger points in neck and shoulder-related disorders: a systematic review of the literature
Source: BMC Musculoskelet Disord. 2018 Jul 25;19:252. doi: 10.1186/s12891-018-2157-9 (PMC6060458; doi:10.1186/s12891-018-2157-9)
Supplement: Supplementary file 1 — Protocol. (DOCX 94 kb) [file 12891_2018_2157_MOESM1_ESM.docx]

**The prevalence of myofascial trigger points in neck and shoulder-related disorders: protocol of systematic review of the literature**

# Introduction

Neck and shoulder pain are common complaints that may significantly impact a person’s activities of daily living and their ability to work [1]. In New Zealand, shoulder pain is the third most common musculoskeletal condition. It accounted for 81,000 medical fees under Accident Compensation Corporation (ACC) in 2002, with the total annual cost for shoulder injuries amounting to 37 million dollars [2]. Neck pain is a leading cause of years lived with disability. Within New Zealand, ACC reports the 12-month prevalence estimates for neck pain in the adult population lie between 30% and 50%, and accounts for 15% of the global burden of disease [3-5].

Myofascial trigger points (MTrPs) are hypersensitive, tender areas over a taut band of muscle [6]. They are palpable, produce localised and referred pain to other structures with mechanical stimulation [7, 8]. MTrPs can be further differentiated as active or latent [6]. Active and latent MTrPs elicit local and referred pain, however active MTrPs also reproduce patient symptoms, whereas latent MTrPs do not [6, 7, 9]. Latent MTrPs may later become active [6, 10]. It is considered that both active and latent MTrPs can cause muscle imbalances, weakness and impaired motor recruitment, disrupting muscle function, and exposing joint to suboptimal loading [11].

Active and latent MTrPs can contribute to neck and shoulder pain symptoms [12]. Active MTrPs present higher concentration of inflammatory mediators, neuropeptides, cytokines, and catecholamines if compared to latent MTrPs or other body regiions with no MTrPs [13]. Patients with chronic, non-traumatic neck and shoulder pain have a higher prevalence of MTrPs when compared to healthy controls, with different distributions between muscles of two opposing anatomical structures [12]. For example, active MTrPs were prevalent in the infraspinatus and upper trapezius muscles, whilst latent MTrPs were prevalent in the teres major and anterior deltoid muscles [12]. Studies investigating shoulder impingement have reported active MTrPs in infraspinatus, subscapularis, supraspinatus, and pectoralis major muscles [14]. This indicates that, although MTrPs are present in different shoulder and neck disorders, they vary in muscle distribution and type (i.e. active or latent).

Knowledge of MTrP common locations at the neck and shoulder can help clinicians to optimally prescribe interventions to manage neck and shoulder disorders. To our knowledge no previous systematic review summarized findings from and assessed the methodological quality of studies assessing the prevalence of MTrPs in neck and shoulder disorders. Therefore, the objective of this study is to synthesize the current evidence on the prevalence of active and latent MTrPs in subjects with neck and shoulder disorders. The specific aims are to: (1) identify the prevalence of MTrPs in scapular and shoulder muscles; and (2) compare the prevalence of MTrPs in subjects with diagnosed neck or shoulder-related disorders to healthy controls.

# Methods

## Study retrieval and screening

We will conduct a comprehensive literature search of databases including CINAHL, Embase, Pubmed, Scopus and Web of Science. We will also screen reference lists from included studies. We will screen retrieved articles for eligibility by title, followed by full-article screening.

Initially, two independent reviewers will screen articles by titles, and a third reviewer will be available if consensus is not achieved. Full texts of potential eligible studies will be retrieved and assessed independently against the inclusion criteria by two reviewers. Discrepancies between reviewers regarding full text eligibility will be resolved in a consensus meeting and a third reviewer will be consulted.

Eligibility criteria

The following study designs will be included in this review: (1) full-text articles published in a peer-reviewed scientific journal; (2) observational, cross-sectional, or prospective studies assessing the prevalence of active and/or latent MTrPs in at least one group of adult subjects (> 18 years old) with a shoulder, scapular, or neck disorder; and (3) inclusion of manual assessment of MTrPs in at least one specific neck, scapular or shoulder muscle.

Articles in all languages and medical diagnoses indicating the presence of shoulder, scapular, or neck will be included in this review. All study designs other than the aforementioned will be excluded. We will include randomised control trials if the prevalence of MTrPs as a baseline measurement is reported.

## Risk of bias within included studies

A modified Downs and Black checklist (Downs and Black 1998) will be used to assess the risk of bias within included studies. Two reviewers will assess each study independently. Disagreements will be resolved through consensus, if consensus is not reached, then a third author (D.R) will be consulted. Studies scoring 50% or more will be considered as having low risk of bias; whilst studies presenting with a Downs and Black score lower than 50% will be considered as having a high risk of bias.

## Data extraction

Characteristics from each study and additional patient and control group information will be extracted and recorded. The proportion of participants with active/latent MTrPs in all assessed muscles will be documented from each study. The data will be independently extracted by two reviewers, and double-checked for accuracy.

## Data analysis

We will conduct meta-analysis if we have sufficient data. Otherwise, a narrative discussion of findings will be presented.

## Ethical Approval

Not applicable.

## Funding

No funding was received to conduct this review.

## Conflict of Interest

The authors declare no conflict of interest.

## References

1. Greenberg, D.L., *Evaluation and treatment of shoulder pain.* Medical Clinics of North America, 2014. **98**(3): p. 487-504.

2. Arroll B, et al. *The diagnosis and management of soft tissue shoulder injuries and related disorders.* 2004; Available from: <http://www.acc.co.nz/PRD_EXT_CSMP/groups/external_communications/documents/guide/wcm001684.pdf>.

3. ACC. *Acute Neck Pain.* 2009; Available from: <http://www.acc.co.nz/1>. PRD_EXT_CSMP/groups/external_providers/documents/guide/prd_ctrb110719.pdf.

4. Vos, T., et al., *Global, regional, and national incidence, prevalence, and years lived with disability for 301 acute and chronic diseases and injuries in 188 countries, 1990-2013: A systematic analysis for the Global Burden of Disease Study 2013.* The Lancet, 2015. **386**(9995): p. 743-800.

5. Vos, T., et al., *Years lived with disability (YLDs) for 1160 sequelae of 289 diseases and injuries 1990-2010: A systematic analysis for the Global Burden of Disease Study 2010.* The Lancet, 2012. **380**(9859): p. 2163-2196.

6. Irnich, D., *Myofascial Trigger Points: Comprehensive diagnosis and treatment*. Myofascial Trigger Points: Comprehensive Diagnosis and Treatment. 2013: Elsevier Ltd. 1-568.

7. Rha, D.W., et al., *Detecting local twitch responses of myofascial trigger points in the lower-back muscles using ultrasonography.* Arch Phys Med Rehabil, 2011. **92**(10): p. 1576-1580 e1.

8. Alonso-Blanco, C., et al., *Prevalence and Anatomical Localization of Muscle Referred Pain from Active Trigger Points in Head and Neck Musculature in Adults and Children with Chronic Tension-Type Headache.* Pain Medicine, 2011. **12**(10): p. 1453-1463.

9. Shah, J.P., et al., *Myofascial Trigger Points Then and Now: A Historical and Scientific Perspective.* PM R, 2015. **7**(7): p. 746-61.

10. Celik, D. and E.K. Mutlu, *Clinical implication of latent myofascial trigger point.* Curr Pain Headache Rep, 2013. **17**(8): p. 353.

11. Castaldo, M., et al., *Myofascial trigger points in patients with whiplash-associated disorders and mechanical neck pain.* Pain Medicine (United States), 2014. **15**(5): p. 842-849.

12. Liu, L., et al., *Effectiveness of dry needling for myofascial trigger points associated with neck and shoulder pain: A systematic review and meta-analysis.* Archives of Physical Medicine and Rehabilitation, 2015. **96**(5): p. 944-955.

13. Shah, J.P., et al., *Biochemicals Associated With Pain and Inflammation are Elevated in Sites Near to and Remote From Active Myofascial Trigger Points.* Archives of Physical Medicine and Rehabilitation, 2008. **89**(1): p. 16-23.

14. Hidalgo-Lozano, A., et al., *Changes in pain and pressure pain sensitivity after manual treatment of active trigger points in patients with unilateral shoulder impingement: A case series.* Journal of Bodywork and Movement Therapies, 2011. **15**(4): p. 399-404.
